# Cannabidiol Enhances the Therapeutic Efficacy of Olsalazine and Cyclosporine in a Murine Model of Colitis

**DOI:** 10.3390/ijms26167913

**Published:** 2025-08-16

**Authors:** Dinesh Thapa, Mohan Patil, Leon N. Warne, Rodrigo Carlessi, Marco Falasca

**Affiliations:** 1 Curtin Medical Research Institute, Curtin University, Perth, WA 6102, Australia; mohanpatilpharmacology@gmail.com (M.P.); leon.warne@anaesthesia.vet (L.N.W.); rodrigo.carlessi@curtin.edu.au (R.C.); 2 College of Science, Health, Engineering and Education, Murdoch University, Perth, WA 6150, Australia; 3 Harry Perkins Institute of Medical Research, QEII Medical Centre for Medical Research, The University of Western Australia, Nedlands, WA 6009, Australia; 4 Department of Medicine and Surgery, University of Parma, 43125 Parma, Italy; 5 Molecular Endocrinology and Pharmacology, Harry Perkins Institute of Medical Research and Centre for Medical Research, The University of Western Australia, Nedlands, WA 6009, Australia

**Keywords:** cannabidiol, IBD, DSS-induced colitis, olsalazine, cyclosporine, combination therapy, inflammation, cytokines

## Abstract

Current therapies for inflammatory bowel disease (IBD), such as olsalazine and cyclosporine, often exhibit limited long-term efficacy and are associated with adverse effects. Cannabidiol (CBD), a non-psychoactive phytocannabinoid, shows promise for its anti-inflammatory properties, though its effectiveness as a monotherapy remains inconclusive. This study investigates the therapeutic potential of combining low-dose CBD (10 mg/kg) with olsalazine (50 mg/kg) or cyclosporine (2.5, 5 mg/kg) in dextran sulphate sodium (DSS)-induced acute and chronic colitis models in mice. Disease severity was assessed via disease activity index (DAI), colon morphology, cytokine and chemokine expression, myeloperoxidase (MPO) activity, systemic inflammatory markers, and glucagon-like peptide-1 (GLP-1) regulation. Safety evaluations included haematology and plasma biochemistry. DSS-treated mice showed elevated DAI scores, colon shortening, heightened inflammation, and organ enlargement. Combination therapies significantly ameliorated colitis, reducing DAI, MPO activity, and inflammatory cytokines, while restoring colon length and GLP-1 levels—without inducing liver or kidney toxicity. These findings demonstrate that combining a low dose of CBD with standard IBD drugs enhances therapeutic efficacy while minimizing side effects, supporting its integration into future combination strategies for more effective and safer IBD management.

## 1. Introduction

Inflammatory bowel disease (IBD), comprising ulcerative colitis (UC) and Crohn’s disease (CD), affects millions of individuals worldwide, imposing significant health and economic burdens [1]. IBD is a major global health challenge affecting nearly 5 million people worldwide, with persistent growth in total cases and notable regional variations—surging in industrialized nations and rapidly increasing in developing countries [2,3]. Characterized by chronic inflammation of the gastrointestinal tract, IBD manifests as abdominal pain, diarrhoea, weight loss, and rectal bleeding. Although the precise aetiology of IBD remains unclear, it is widely recognized as a multifactorial disease involving genetic predisposition, environmental triggers, immune dysregulation, and alterations in the gut microbiota [4,5,6,7,8].

Current therapeutic approaches for IBD, including aminosalicylates, corticosteroids, immunosuppressants, and biologic agents targeting cytokines like tumour necrosis factor-α (TNF-α), have improved disease management [9,10]. Despite the availability of various therapeutic options, a significant proportion of patients with IBD fail to achieve sustained remission or experience adverse effects, underscoring the urgent need for novel and safer treatment strategies [5,11,12]. In recent years, growing attention has been directed toward natural products with anti-inflammatory and immunomodulatory properties as potential therapeutic agents [13]. To overcome the current plateau in treatment efficacy, rational combination therapies that engage complementary mechanisms of action are being explored. Such strategies require a deeper understanding of drug interactions, mucosal immunology, and host–microbiome dynamics [14]. Notably, a clinical trial (NCT01320436) demonstrated that the addition of curcumin to standard mesalamine therapy was significantly more effective than mesalamine alone in inducing both clinical and endoscopic remission in patients with mild-to-moderate ulcerative colitis, without any apparent adverse effects [15].

Cannabidiol (CBD), a non-psychoactive phytocannabinoid derived from *Cannabis sativa*, has emerged as a promising therapeutic candidate for the treatment of inflammatory conditions, including IBD [16,17]. CBD’s anti-inflammatory, antioxidant, and immunomodulatory effects are mediated through multiple pathways, including the modulation of cytokine production, inhibition of oxidative stress, and interaction with the endocannabinoidome (eCBome) [18,19]. The eCBome is a complex endogenous lipid-signalling network that plays a pivotal role in maintaining gut homeostasis by regulating immune responses, epithelial barrier function, and gut motility [20]. Dysregulation of the eCBome has been implicated in the pathogenesis of IBD, making it an attractive target for therapeutic intervention [20,21,22]. Modulation of the eCBome through cannabinoids like CBD has shown potential in reducing intestinal inflammation and promoting mucosal healing [16,23,24]. Preclinical studies have demonstrated CBD’s efficacy in reducing inflammation and tissue damage in experimental models of colitis [16,25,26]. Additionally, CBD has shown a favourable safety profile in both animal and human studies, making it an attractive therapeutic option [16,27].

Cyclosporine, a calcineurin inhibitor, and olsalazine, a prodrug of mesalamine, are well-established agents for the management of IBD. Cyclosporine’s potent immunosuppressive properties make it effective in severe cases of UC, while olsalazine’s ability to deliver mesalamine directly to the colon has proven beneficial in mild to moderate UC [28]. Recently, olsalazine has also been shown to exert its effects by activating G protein-coupled receptor 35 (GPR35), a member of the eCBome that is highly expressed in the gastrointestinal tract and plays a crucial role in modulating inflammation and maintaining epithelial integrity [29]. However, therapeutic doses of olsalazine and cyclosporine often present with significant adverse effects, such as nephrotoxicity and gastrointestinal disturbances [30], underscoring the need for strategies that enhance their efficacy while minimizing adverse effects.

Emerging evidence suggests that combination therapies using lower doses of conventional drugs alongside complementary agents such as cannabidiol may yield superior therapeutic outcomes across a range of diseases, including cancer [31,32,33]. These strategies aim to harness synergistic interactions that enhance anti-inflammatory effects while reducing the adverse effects often associated with high-dose monotherapies. Among the key mediators of such synergy is the endocannabinoid system (ECS), particularly the cannabinoid receptor type 1 (CB1) and cannabinoid receptor type 2 (CB2), which are increasingly recognized for their roles beyond nociceptive (pain) modulation. Notably, activation of these receptors influences critical intracellular signalling pathways, including mitogen-activated protein kinase (MAPK), phosphoinositide 3-kinase/protein kinase B (PI3K/Akt), nuclear factor kappa-light-chain-enhancer of activated B cells (NF-κB), and the nucleotide-binding oligomerization domain-like receptor protein 3 (NLRP3) inflammasome—pathways integral to immune cell activation, cytokine production, and epithelial barrier function [21,34,35,36,37]. Activation of these receptors has been shown to modulate the MAPK, PI3K/Akt, and NF-κB pathways, as well as the NLRP3 inflammasome, all of which are critically involved in immune cell activation, cytokine release, and epithelial barrier function [35,36,37]. Importantly, many of these pathways also overlap with those targeted by conventional therapies used in inflammatory bowel disease (IBD), suggesting that cannabinoids may enhance treatment outcomes through receptor crosstalk and convergent intracellular signalling. This mechanistic overlap underscores the translational potential of cannabinoid-based combination therapies in IBD [38,39,40]. However, despite promising findings in cancer [31], there remains a lack of comprehensive preclinical studies evaluating the efficacy and safety of combining CBD with existing IBD therapies.

While conventional therapies for inflammatory bowel disease (IBD) primarily focus on modulating pro-inflammatory cytokines such as TNF-α, emerging evidence highlights the involvement of glucagon-like peptide-1 (GLP-1)—an incretin hormone with anti-inflammatory and cytoprotective properties—in the pathogenesis and potential treatment of IBD [41,42]. Recent studies have demonstrated that CBD, either alone or in combination with tetrahydrocannabinol (THC), can restore physiological GLP-1 levels in murine models of colitis [16,43], suggesting a broader therapeutic role for cannabinoids in modulating gut hormone pathways and inflammation in colitis.

This study aimed to investigate the therapeutic potential of combining sub-therapeutic doses of cannabidiol with either cyclosporine or olsalazine in a dextran sulphate sodium (DSS)-induced mouse model of colitis. The DSS model, widely used due to its close resemblance to human inflammatory bowel disease (IBD), enables comprehensive evaluation of disease activity, histopathological alterations, and inflammatory responses. Key parameters assessed included disease activity index (DAI), colon length, myeloperoxidase (MPO) activity, cytokine and chemokine profiles, and glucagon-like peptide-1 (GLP-1) levels, alongside haematological and biochemical markers to evaluate treatment safety. To the authors’ knowledge, this is the first preclinical study to report the synergistic therapeutic effects of low-dose CBD in combination with either cyclosporine or olsalazine in both acute and chronic models of colitis. These findings provide novel insights into the efficacy and safety of CBD-based combination therapies and support their potential as optimized treatment strategies for IBD.

## 2. Results

### 2.1. Cannabidiol Combined with Cyclosporine A or Olsalazine Alleviates Clinical Signs in Acute Colitis

**Figure 1:**
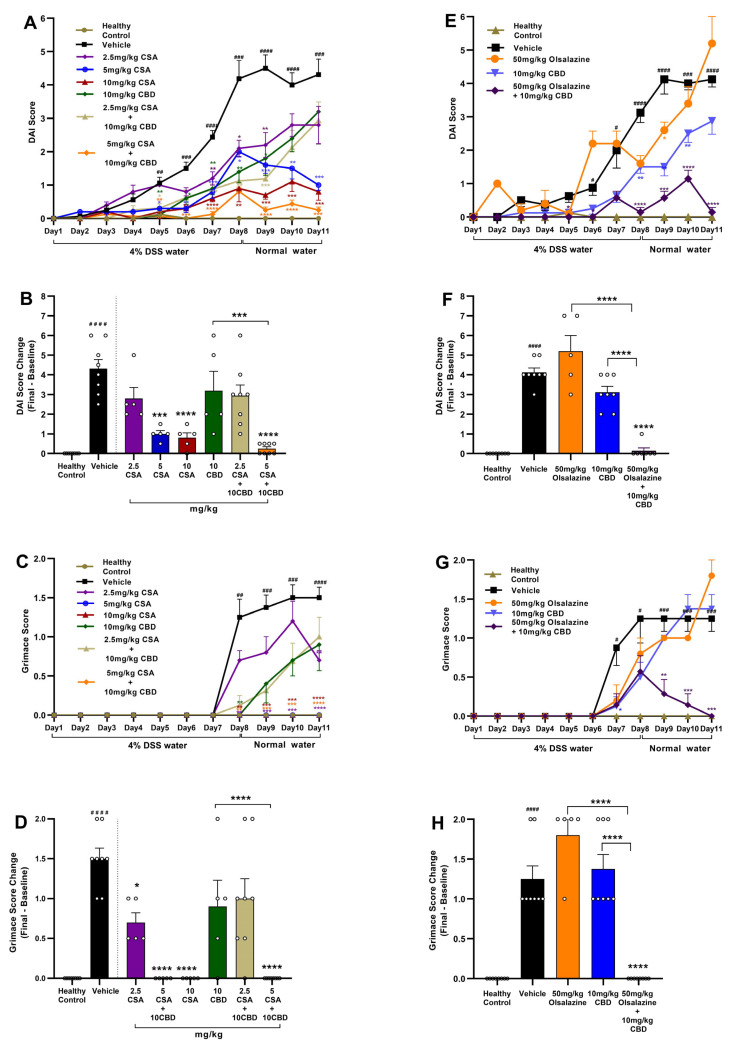
Combination therapy with CBD and CSA or olsalazine alleviates colitis severity and visceral pain. (**A**,**E**) Time-course of DAI scores during DSS and recovery phases for CSA and olsalazine studies, respectively. (**B**,**F**) Final change in DAI score from baseline at Day 11. (**C**,**G**) Time-course of mouse grimace scores during DSS and recovery phases. (**D**,**H**) Final change in grimace scores from baseline at Day 11. Data are expressed as mean ± SEM. Statistical significance: # *p* < 0.05, ## *p* < 0.01, ### *p* < 0.001, #### *p* < 0.0001 vs. healthy control; * *p* < 0.05, ** *p* < 0.01, *** *p* < 0.001, **** *p* < 0.0001 vs. vehicle.

To assess the therapeutic efficacy of CBD in combination with CSA or olsalazine, DAI and pain scores were evaluated in DSS-induced acute colitis mice. In the CSA treatment cohort (Figure 1A,B), CSA monotherapy produced a dose-dependent reduction in DAI scores, with the greatest effect observed at 10 mg/kg compared to the vehicle control (*p* < 0.0001). In contrast, CBD administered alone at 10 mg/kg showed only a transient effect and failed to sustain DAI improvement through the study endpoint. Notably, the combination of 10 mg/kg CBD with 5 mg/kg CSA significantly reduced DAI scores beginning on day 5, with the effect sustained through the end of the study (*p* < 0.0001). The combination treatments also demonstrated a superior efficacy compared to CBD alone (*p* < 0.001).

Visceral pain was assessed using the mouse grimace score (Figure 1C,D). A significant increase in grimace scores was observed in the vehicle-treated colitis group compared to healthy controls beginning on day 7 (*p* < 0.01), which persisted through the study endpoint (*p* < 0.0001). While CSA monotherapy at both 5 mg/kg and 10 mg/kg significantly reduced grimace scores compared to the vehicle group, CBD monotherapy at 10 mg/kg did not produce a significant effect. Notably, the combination of 10 mg/kg CBD with 5 mg/kg CSA resulted in a near-complete suppression of grimace scores by the study endpoint (*p* < 0.0001). 5 mg/kg CSA also potentiated the efficacy of 10 mg/kg CBD.

In the olsalazine treatment cohort (Figure 1E,F), olsalazine administered alone at 50 mg/kg failed to reduce DAI scores, and CBD monotherapy at 10 mg/kg produced only a transient improvement that was not sustained through the study endpoint compared to vehicle control. In contrast, the combination of 10 mg/kg CBD with 50 mg/kg olsalazine significantly attenuated DAI progression and markedly reduced disease severity by the study endpoint (*p* < 0.0001; Figure 1F). Similarly, visceral pain, as assessed by grimace scoring, was significantly reduced in the combination group (Figure 1G,H), compared to the vehicle group. The combination treatments also showed superior efficacy compared to monotherapies.

Together, these results indicate that low-dose combination therapies involving CBD and either CSA or olsalazine reduce disease severity and pain in chronic colitis by potentiating the efficacy of monotherapies.

### 2.2. Cannabinoid-Based Combinatorial Therapy Ameliorates Clinical Signs in Chronic Colitis

The most effective cannabinoid-based combination therapy obtained from the acute study was evaluated in a chronic model of DSS-induced colitis. In the chronic study, key clinical parameters, including DAI, body weight, diarrhoea, and faecal blood scores, were assessed across treatment groups.

**Figure 2:**
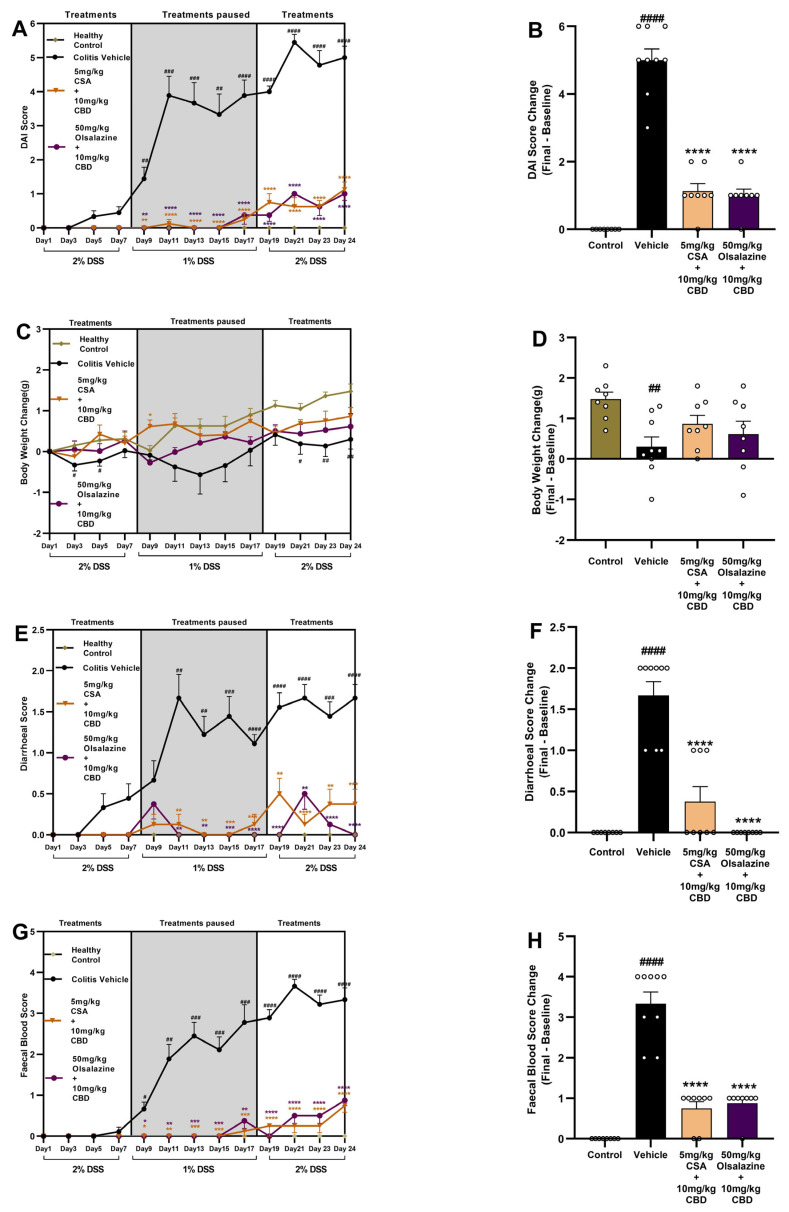
Cannabinoid-based combination therapy attenuates clinical disease parameters in chronic DSS-induced colitis. Mice received 2% DSS from days 1–7, 1% DSS from days 8–17 (treatment pause, shaded), and 2% DSS from days 18–24. Panels (**A**,**C**,**E**,**G**) show time courses of DAI, body weight change, diarrhoea score, and faecal blood score, respectively; panels (**B**,**D**,**F**,**H**) depict final changes from baseline at day 24. Data are presented as mean ± SEM. Statistical significance: # *p* < 0.05, ## *p* < 0.01, ### *p* < 0.001, #### *p* < 0.0001 vs. healthy control; and * *p* < 0.05, ** *p* < 0.01, *** *p* < 0.001, **** *p* < 0.0001 vs. vehicle.

Administration of DSS induced a significant increase in endpoint DAI scores in the colitis vehicle group (5 ± 0.3) compared to healthy controls (0 ± 0, *p* < 0.001, Figure 2B). In contrast, mice receiving combination therapy of 10 mg/kg CBD + 5 mg/kg CSA or 10 mg/kg CBD + 50 mg/kg olsalazine exhibited significant reductions in DAI scores throughout the treatment period, with therapeutic effects sustained even during the treatment interruption. Final DAI score changes from baseline further confirmed significant attenuation in the combination treatment groups (1.1 ± 0.2 and 1.0 ± 0.2, respectively) compared to the vehicle group (*p* < 0.0001; Figure 2B,F). The mean DAI score of the combination group is similar to that of the healthy control group.

At the study endpoint, the vehicle group exhibited significantly greater body weight loss compared to healthy controls (*p* < 0.01; Figure 2D). A significant improvement in body weight was observed on day 9 in the 5 mg/kg CSA and 10 mg/kg CBD-treated group compared to the vehicle group (*p* < 0.05; Figure 2C). However, by the study endpoint, no significant differences in body weight were observed between the vehicle group and either of the combination treatment groups (5 mg/kg CSA + 10 mg/kg CBD or 50 mg/kg olsalazine + 10 mg/kg CBD; Figure 2D).

Diarrhoeal scores increased substantially in vehicle-treated groups compared to healthy controls (*p* < 0.0001; Figure 2E,F). At the study endpoint, both cannabinoid-based combination therapies significantly reduced diarrhoea severity, with consistent suppression across all treatment phases (*p* < 0.0001 vs. vehicle; Figure 2F). Similarly, faecal blood scores were elevated in the vehicle-treated colitis group (Figure 2G), while combination therapies resulted in significant suppression of faecal blood. The final faecal blood score change was significantly reduced in both CSA + CBD and olsalazine + CBD groups (*p* < 0.0001 vs. vehicle; Figure 2H).

Collectively, these findings demonstrate that both cyclosporine A + CBD and olsalazine + CBD combinations provide robust clinical improvement in a chronic DSS model, with significant effects on DAI, body weight maintenance, and reduction in diarrhoea and faecal blood loss.

### 2.3. Cannabidiol Combination Therapies Restore Colon Length and Reduce Local and Systemic Inflammation in Acute Colitis

**Figure 3:**
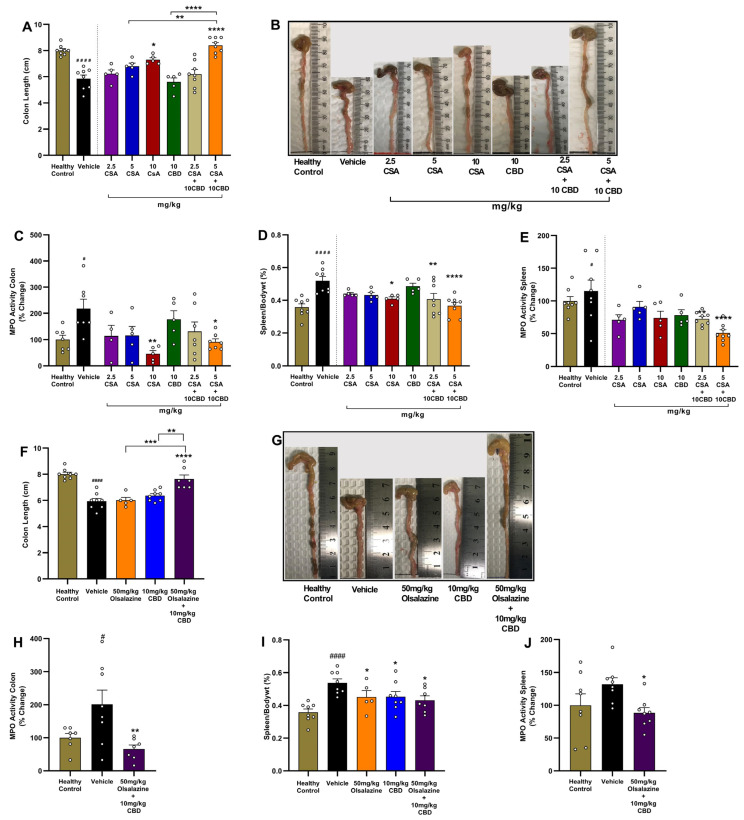
Low-dose CBD co-therapies restore colon length and suppress neutrophilic and splenic inflammation. Panels (**A**–**E**) show the CSA study; (**F**–**J**) show the olsalazine study. (**A**,**F**) Terminal colon length (cm) measured on Day 11. (**B**,**G**) Representative photographs of colons from each group. (**C**,**H**) Colonic MPO activity expressed as % of healthy or vehicle controls. (**D**,**I**) Spleen index (spleen weight/body weight × 100%). (**E**,**J**) Splenic MPO activity expressed as % of healthy or vehicle controls. Data are mean ± SEM. Statistical significance: # *p* < 0.05, #### *p* < 0.0001 vs. healthy control; * *p* < 0.05, ** *p* < 0.01, *** *p* < 0.001, **** *p* < 0.0001 vs. vehicle.

In the CSA treatment cohort, the vehicle group had a significant reduction in colon length compared to healthy controls (*p* < 0.0001; Figure 3A). While monotherapy with CSA at a 10 mg/kg dose significantly increased colon length compared to the vehicle group (*p* < 0.01), 10 mg/kg CBD or 5 mg/kg CSA alone did not increase colon length. In contrast, the combination of 10 mg/kg CBD with 5 mg/kg CSA resulted in a significant increase in colon length compared to the vehicle group (*p* < 0.0001) and demonstrated superior efficacy compared to monotherapies.

Myeloperoxidase (MPO) activity in colon tissue, a surrogate of neutrophil infiltration, was elevated ~2-fold in vehicle-treated colitis mice (*p* < 0.05; Figure 3C). Monotherapies were not effective in reducing MPO activity except for the highest dose of CSA, i.e., 10 mg/kg (*p* < 0.01 vs. vehicle). Interestingly, the combination of 5 mg/kg CSA and 10 mg/kg CBD significantly reduced colon MPO activity (*p* < 0.05 vs. vehicle). The highest CSA dose (10 mg/kg) administered alone also significantly reduced colonic MPO activity (*p* < 0.01).

Systemic inflammation was evaluated using relative spleen-to-body weight percentage and splenic MPO activity. Vehicle group exhibited significant splenomegaly and elevated MPO activity compared to healthy controls (*p* < 0.0001 and *p* < 0.05, respectively) While monotherapies with CBD or lower dose CSA were largely ineffective, combination treatment with 10 mg/kg CBD and either 2.5 mg/kg or 5 mg/kg CSA significantly reduced relative spleen weight compared to the vehicle group (*p* < 0.01 and *p* < 0.0001, respectively; Figure 3D). CSA monotherapy at a higher dose, 10 mg/kg, also significantly decreased spleen weight (*p* < 0.05). Additionally, combination treatment with 10 mg/kg CBD and either 2.5 mg/kg or 5 mg/kg CSA significantly reduced Splenic MPO activity compared to the vehicle group (*p* < 0.01 and *p* < 0.0001, respectively; Figure 3E).

In the olsalazine cohort, similar benefits were observed. While monotherapy of 50 mg/kg olsalazine or 10 mg/kg CBD did not significantly affect colon length, treatment with the combination of 50 mg/kg olsalazine and 10 mg/kg CBD significantly increased colon length compared to the vehicle group (*p* < 0.0001; Figure 3F,G). The effect demonstrated by combination therapies was superior to monotherapies. The combination treatment also significantly reduced colonic MPO activity (*p* < 0.01; Figure 3H). Relative spleen weight and splenic MPO activity were also significantly reduced by combination therapy compared to the vehicle group (*p* < 0.05; Figure 3I,J).

Together, these data demonstrate that low-dose CBD co-therapy with CSA or olsalazine not only attenuates clinical disease activity but also preserves colon integrity and reduces both local and systemic inflammatory markers in acute colitis.

### 2.4. Combination Therapy Preserves Colon Length and Reduces MPO Activity in Colon and Spleen in Chronic Colitis

To evaluate the impact of cannabinoid-based combination therapy on gross intestinal and systemic inflammation, we measured colon length, MPO activity in colon tissue, spleen-to-body weight ratio, and splenic MPO activity at study end (Day 24).

**Figure 4:**
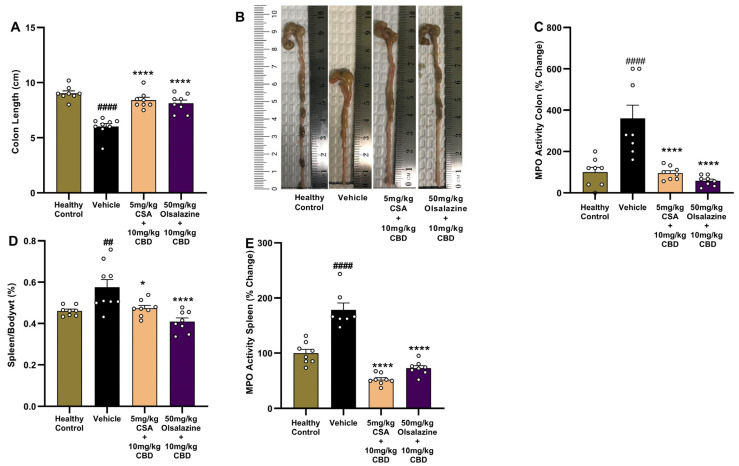
Gross and biochemical markers of colon and systemic inflammation following chronic DSS-induced colitis. (**A**) Colon length. (**B**) Representative images of excised colons from each group. (**C**) Colonic MPO activity expressed as percentage change from healthy control or colitis control. (**D**) Spleen-to-body weight ratio (%). (**E**) Splenic MPO activity expressed as percentage change from healthy control or vehicle control. Data represent mean ± SEM. Statistical significance: ## *p* < 0.01, #### *p* < 0.0001 vs. healthy control; * *p* < 0.05, **** *p* < 0.0001 vs. vehicle.

DSS-induced chronic colitis significantly shortened colon length in vehicle-treated mice compared to healthy controls (6.0 ± 0.3 vs. 9.0 ± 0.2; *p* < 0.0001, Figure 4A,B). In contrast, both 5 mg/kg cyclosporine A + 10 mg/kg CBD and 50 mg/kg olsalazine + 10 mg/kg CBD combinations significantly increased colon length compared to the vehicle group (8.4 ± 0.3 vs. 8.1 ± 0.3; *p* < 0.0001). The mean colon length of the combination group is similar to that of the healthy control group.

Colonic MPO activity was significantly increased in vehicle-treated mice compared to the healthy control (*p* < 0.0001; Figure 4C). In contrast, both combination treatments significantly reduced MPO activity compared to the vehicle group (*p* < 0.0001).

In parallel, DSS markedly increased splenic weight relative to body weight in the vehicle group compared to the healthy control (*p* < 0.01; Figure 4D). Both combination treatments, CBD + CSA or CBD + olsalazine, significantly reduced the relative spleen weight percentage compared to the vehicle group (*p* < 0.05 and *p* < 0.0001, respectively).

Splenic MPO activity was elevated in the vehicle group (*p* < 0.0001 vs. control; Figure 4E). Both treatments reduced splenic MPO activity compared to the vehicle group (*p* < 0.0001), indicating attenuation of systemic inflammatory burden.

Overall, these results demonstrate that cannabinoid-based combinations preserve colon architecture, suppress neutrophil infiltration in both colon and spleen, and limit splenomegaly, underscoring their protective effects in chronic DSS-induced colitis.

### 2.5. Low-Dose Cannabidiol Combination Therapy Inhibits Pro-Inflammatory Cytokine Expression in Acute Colitis

**Figure 5:**
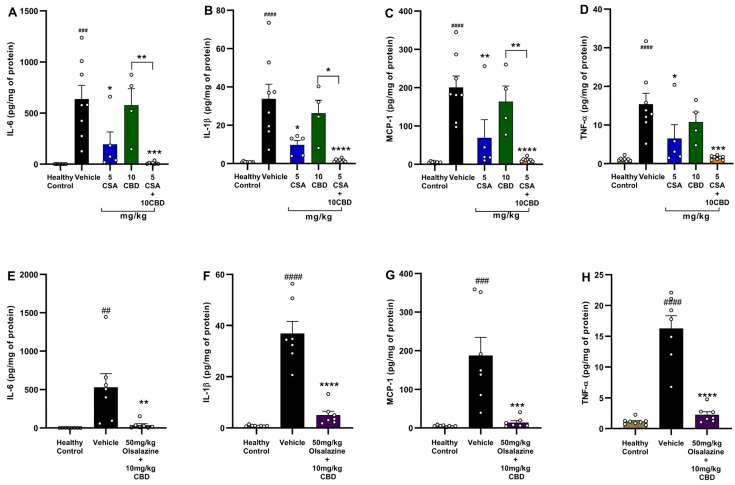
Combination therapy with CBD and CSA or olsalazine abolishes colonic pro-inflammatory cytokine expression in acute colitis. (**A**–**D**) CSA study: colonic concentrations of (**A**) IL-6, (**B**) IL-1β, (**C**) MCP-1, and (**D**) TNF-α (pg/mg protein) in healthy control, colitis vehicle, 5 mg/kg CSA, 10 mg/kg CBD, and 5 mg/kg CSA + 10 mg/kg CBD groups. (**E**–**H**) Olsalazine study: colonic concentrations of (**E**) IL-6, (**F**) IL-1β, (**G**) MCP-1, and (**H**) TNF-α in healthy control, colitis vehicle, 50 mg/kg olsalazine, 10 mg/kg CBD, and 50 mg/kg olsalazine + 10 mg/kg CBD groups. Data are expressed as mean ± SEM. Statistical significance: ## *p* < 0.01, ### *p* < 0.001, #### *p* < 0.0001 vs. healthy control; * *p* < 0.05, ** *p* < 0.01, *** *p* < 0.001, **** *p* < 0.0001 vs. vehicle.

To evaluate local anti-inflammatory effects, colonic levels of IL-6, IL-1β, MCP-1, and TNF-α were quantified at Day 11 in DSS-induced chronic colitis mice (Figure 5). In the CSA cohort (Panels A–D), vehicle-treated colitis mice exhibited marked elevations in IL-6 (*p* < 0.001), IL-1β (*p* < 0.0001), MCP-1 (*p* < 0.0001), and TNF-α (*p* < 0.0001) compared to healthy controls. Monotherapy with 5 mg/kg CSA produced modest reductions in IL-6 and MCP-1 (both *p* < 0.05), while 10 mg/kg CBD had no significant reductions. Strikingly, the 5 mg/kg CSA + 10 mg/kg CBD combination significantly reduced IL-6 (*p* < 0.001), IL-1β (*p* < 0.0001), MCP-1 (*p* < 0.0001), and TNF-α (*p* < 0.001) compared to the vehicle group. Moreover, 5 mg/kg CSA potentiated the anti-inflammatory effects of 10 mg/kg CBD as observed in IL-6 (*p* < 0.01), IL-1β (*p* < 0.05), and MCP-1 (*p* < 0.01).

In the olsalazine cohort (Panels E–H), vehicle mice again showed elevated IL-6 (*p* < 0.01), IL-1β (*p* < 0.0001), MCP-1 (*p* < 0.001), and TNF-α (*p* < 0.0001) compared to healthy controls. Combination treatment with 50 mg/kg olsalazine + 10 mg/kg CBD significantly reduced IL-6 (*p* < 0.01), IL-1β (*p* < 0.0001), MCP-1 (*p* < 0.001), and TNF-α (*p* < 0.0001) compared to vehicle.

These findings demonstrate that low-dose CBD synergizes with CSA or olsalazine to effectively attenuate colonic pro-inflammatory cytokine production in chronic DSS colitis.

### 2.6. Combination Therapy with Lower Dose of Cannabidiol, Olsalazine, and Cyclosporine Attenuates Pro-Inflammatory Cytokine Expression in Chronic Colitis

**Figure 6:**
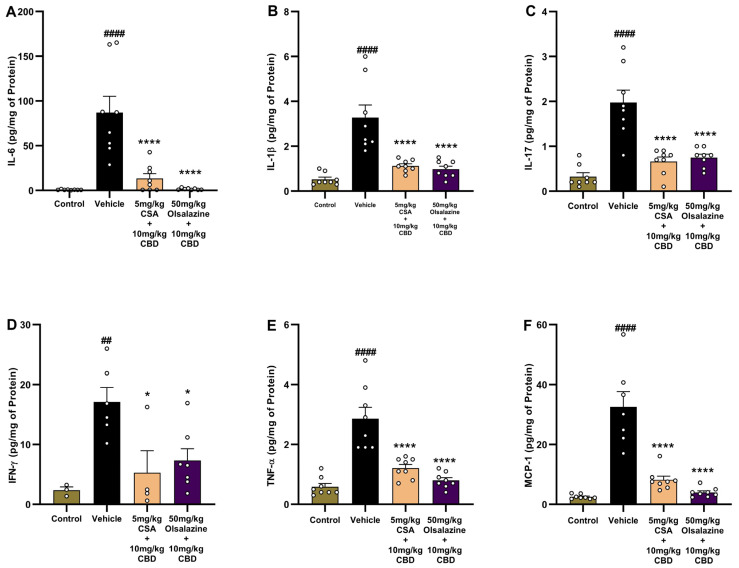
Combination therapy with low-dose CBD, olsalazine, or cyclosporine A reduces pro-inflammatory cytokines in chronic colitis. Colonic levels of IL-6 (**A**), IL-1β (**B**), IL-17 (**C**), IFN-γ (**D**), TNF-α (**E**), and MCP-1 (**F**) in chronic colitis mice treated with vehicle, CBD (10 mg/kg) + CSA (5 mg/kg), or CBD (10 mg/kg) + olsalazine (50 mg/kg). Data are expressed as mean ± SEM. Statistical significance: ## *p* < 0.01, #### *p* < 0.0001 vs. healthy control; * *p* < 0.05, **** *p* < 0.0001 vs. vehicle.

Key pro-inflammatory cytokines in colonic tissue were quantified to evaluate the anti-inflammatory effects of combination therapy, i.e., CBD and olsalazine or CBD and cyclosporine A (CSA), in chronic colitis. As shown in Figure 6A, IL-6 levels were significantly elevated in the vehicle-treated colitis group compared to the healthy control group (0.7 ± 0.1 vs. 87 ± 18; *p* < 0.0001). Treatment with 10 mg/kg CBD + 5 mg/kg CSA or 10 mg/kg CBD + 50 mg/kg olsalazine significantly reduced IL-6 levels (13.4 ± 5.3 and 1.5 ± 0.4, respectively; *p* < 0.0001), indicating strong suppression of inflammation.

Similarly, IL-1β (Figure 6B) and IL-17 (Figure 6C), critical mediators of Th17-driven inflammation, were significantly increased in the vehicle group compared to the healthy control group (0.5 ± 0.1 vs. 3.3 ± 0.5 and 0.3 ± 0.1 vs. 2.0 ± 0.3, respectively; *p* < 0.0001). Both combination treatments (CBD + 5 mg/kg CSA or 10 mg/kg CBD + 50 mg/kg) significantly reduced the levels of IL-1β (1.1 ± 0.1 and 0.1 ± 0.1, respectively) and IL-17 (0.6 ± 0.1 and 0.7 ± 0.1, respectively, *p* < 0.0001), suggesting an effective suppression of Th17-driven immune responses.

IFN-γ (Figure 6D), a marker of Th1-mediated inflammation, was elevated in the vehicle group compared to the healthy control group (2.3 ± 0.5 vs. 17.1 ± 2.4; *p* < 0.01). Both treatments (CBD + 5 mg/kg CSA or 10 mg/kg CBD + 50 mg/kg) significantly reduced IFN-γ levels (5.3 ± 3.6 and 7.3 ± 1.9, respectively; *p* < 0.05) compared to the vehicle-treated group.

TNF-α (Figure 6E) and MCP-1 (Figure 6F) were significantly upregulated in the vehicle-treated colitis group compared to healthy controls (0.6 ± 0.1 vs. 2.9 ± 0.4 and 2.5 ± 0.3 vs. 32.6 ± 5.0, respectively; *p* < 0.0001), highlighting the activation of macrophages and monocyte recruitment in the inflamed colon. Both treatments (CBD + 5 mg/kg CSA or 10 mg/kg CBD + 50 mg/kg) significantly reduced the levels of TNF-α (1.2 ± 0.1 and 0.8 ± 0.1) and MCP-1 (8.1 ± 1.2 and 3.9 ± 0.5, respectively; *p* < 0.0001) to the similar levels of healthy control group, suggesting that both treatments effectively suppressed monocyte/macrophage-driven inflammation.

### 2.7. Cannabidiol Combination Therapy Normalizes Metabolic Parameters, Organ Indices, and GLP-1 Levels in Acute Colitis

**Figure 7:**
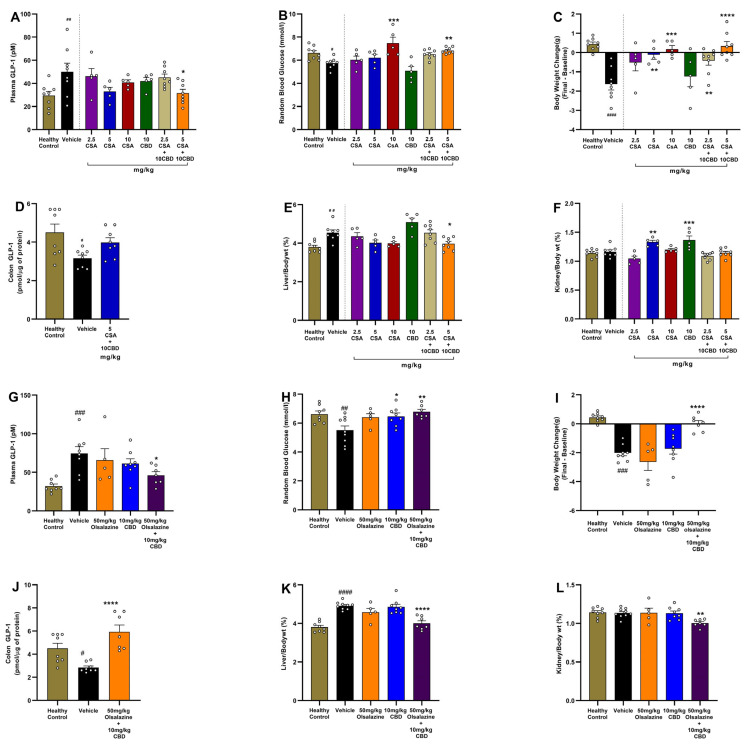
Effects of CBD combination therapies on GLP-1 levels, glycemia, body weight, and organ indices in chronic DSS colitis. CSA study (**A**–**F**): (A) Plasma GLP-1 (pM). (**B**) Random blood glucose (mmol/L). (**C**) Body weight change from baseline (g). (**D**) Colonic GLP-1 content (pmol/mg protein). (**E**) Liver index (liver weight/body weight × 100%). (**F**) Kidney index (kidney weight/body weight × 100%). Olsalazine study (**G**–**L**): (**G**) Plasma GLP-1 (pM). (**H**) Random blood glucose (mmol/L). (**I**) Body weight change from baseline (g). (**J**) Colonic GLP-1 content (pmol/mg protein). (**K**) Relative liver weight (%). (**L**) Relative kidney weight (%). Data are expressed as mean ± SEM. Statistical significance: # *p* < 0.05, ## *p* < 0.01, ### *p* < 0.001, #### *p* < 0.0001 vs. healthy control; * *p* < 0.05, ** *p* < 0.01, *** *p* < 0.001, **** *p* < 0.0001 vs. vehicle.

In the CSA cohort (Figure 7, panels A–F), vehicle-treated colitis mice, compared to healthy controls, showed elevated plasma GLP-1 (29.46 ± 3.7 vs. 49.98 ± 7.5; *p* < 0.01; Figure 7A), reduced random blood glucose (6.62 ± 0.21 vs. 5.7 ± 0.2; *p* < 0.001; Figure 7B), increased net body weight loss by Day 11 (0.43 ± 0.11 vs. −1.6 ± 0.29; *p* < 0.0001; Figure 7C), reduced colon GLP-1 (4.5 ± 0.43 vs. 3.16 ± 0.16; *p* < 0.05; Figure 7D), increased relative liver weight (3.8 ± 0.09 vs. 4.54 ± 0.15; *p* < 0.01; Figure 7E) while there was no significant change in relative kidney weight (1.14 ± 0.23 vs. 1.16 ± 0.03; *p* < 0.05; Figure 7F). Monotherapy with 5 mg/kg CSA or 10 mg/kg CBD increased relative kidney weight percentage compared to the vehicle-treated group (1.33 ± 0.03; *p* < 0.01 and 1.36 ± 0.07; *p* < 0.001, respectively, Figure 7F). The combination of 5 mg/kg CSA + 10 mg/kg CBD significantly reduced plasma GLP-1 (31.63 ± 3.3; *p* < 0.05), increased blood glucose (6.8 ± 0.1; *p* < 0.01), increased body weight (0.33 ± 0.23; *p* < 0.0001), and reduced relative liver weight (3.9 ± 0.11; *p* < 0.05) compared to the vehicle group.

In the olsalazine cohort (Figure 7G–L), combination treatment with 50 mg/kg olsalazine and 10 mg/kg CBD reduced plasma GLP-1 (46.16 ± 4.6 vs. 74.47 ± 8.7; *p* < 0.05; Figure 7G), increased blood glucose (6.8 ± 0.16 vs. 6.6 ± 0.2; *p* < 0.01), increased body weight (0.01 ± 0.2 vs. −2.01 ± 0.22; *p* < 0.0001), increased colonic GLP-1 (5.9 ± 0.5 vs. 2.8 ± 0.14; *p* < 0.0001), reduced relative liver weight (4.0 ± 0.13 vs. 4.9 ± 0.06; *p* < 0.0001) and kidney weight 1.0 ± 0.01 vs. 1.13 ± 0.02; *p* < 0.01, respectively). These data indicate that low-dose CBD co-therapies with CSA or olsalazine can correct colitis-associated dysregulation of incretin hormones and metabolic homeostasis.

### 2.8. Cannabinoid-Based Combination Therapy Modulates GLP-1 and Restores Glucose Homeostasis in Chronic Colitis

**Figure 8:**
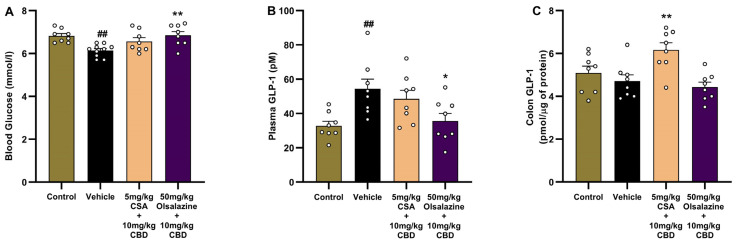
Fasting blood glucose and GLP-1 measurements at Day 24. (**A**) Fasting blood glucose. (**B**) Plasma GLP-1 concentration. (**C**) Colonic GLP-1 concentration. All data are expressed as mean ± SEM. Statistical significance: ## *p* < 0.01 vs. healthy control; * *p* < 0.05, ** *p* < 0.01 vs. vehicle.

To investigate the metabolic and gut-hormonal effects of cannabinoid-based therapy in chronic colitis, we assessed fasting blood glucose, plasma GLP-1, and colonic GLP-1 levels on Day 24. As shown in Figure 8A, vehicle-treated colitis mice exhibited a significant reduction in blood glucose levels compared to the healthy control group (6.8 ± 0.1 vs. 6.1 ± 0.1, *p* < 0.01), consistent with DSS-induced metabolic disturbance. In comparison, no significant reduction in blood glucose level was observed in the CSA + CBD combination compared to the vehicle group. The olsalazine + CBD group showed significantly elevated glucose to a similar level observed in healthy controls (6.8 ± 0.1; *p* < 0.01).

In parallel, plasma GLP-1 levels (Figure 8B) were significantly elevated in vehicle-treated mice compared to the healthy control group (32.8 ± 2.7 vs. 54.3 ± 5.8, *p* < 0.01), suggesting a compensatory endocrine response to inflammation or dysregulated glucose metabolism. In contrast, olsalazine + CBD treatments significantly reduced GLP-1 (35.6 ± 4.4; *p* < 0.05) compared to the vehicle group.

As shown in Figure 8C, while there was no change in colonic GLP-1 levels in the vehicle group compared to the healthy control group, treatments with CSA + CBD significantly increased GLP-1 compared to vehicle-treated mice (6.2 ± 0.3 vs. 4.7 ± 0.3, *p* < 0.01), indicating enhanced local GLP-1 expression. The olsalazine + CBD group showed no significant difference from the vehicle.

Together, these findings demonstrate that both cannabinoid-based combinations restore glucose homeostasis and normalize plasma GLP-1 levels, while CSA + CBD additionally enhances colonic GLP-1, suggesting potential benefits for gut integrity and metabolic regulation in chronic colitis.

### 2.9. Effects of Low-Dose Cannabidiol Combination Therapies on Biochemical Parameters, Organ Indices, and Haematology in Chronic Colitis

To assess the safety of low-dose CBD combination therapies, serum biochemical parameters, relative organ–body weight ratios (Table 1), and comprehensive haematology (Table 2) were assessed at study endpoint (Day 24).

Chronic DSS colitis in vehicle-treated mice induced mild renal and hepatic stress, evidenced by elevated creatinine (*p* < 0.05), hypoalbuminemia (*p* < 0.01), hyperammonemia (*p* < 0.0001), and increased liver (*p* < 0.001) and kidney (*p* < 0.05) relative weights versus healthy controls. Treatments with 5 mg/kg CSA + 10 mg/kg CBD combination had a negligible impact on ALT, AST, BUN, creatinine, TP, globulin, and albumin, but significantly reduced ammonia (*p* < 0.01 vs. vehicle). Liver and kidney indices remained at healthy levels. On the other hand, treatment with a 50 mg/kg olsalazine + 10 mg/kg CBD regimen elevated ALT (*p* < 0.01), AST (*p* < 0.01), and creatinine (*p* < 0.01), indicating mild organ stress. However, it normalized ammonia (*p* < 0.001) and restored relative liver (*p* < 0.001) and kidney (*p* < 0.05) weights compared to the vehicle group.

In Haematological parameters, vehicle-treated colitis mice showed significant decreases in RBC count (*p* < 0.05), haemoglobin (*p* < 0.05), and haematocrit (*p* < 0.05) relative to healthy controls, with no changes in WBC, lymphocytes, monocytes, granulocytes, MCV, MCH, MCHC, RDW, platelets, MPV, PDW, or procalcitonin. No significant change, compared to vehicle, was observed with CSA + CBD combination on all blood indices and remained comparable to healthy groups, indicating no haematological toxicity. However, the olsalazine + CBD group exhibited a significant increase in RBC (*p* < 0.001), haemoglobin (*p* < 0.05), and haematocrit (*p* < 0.05), alongside a marked rise in platelet count (*p* < 0.01) and a decrease in granulocytes (*p* < 0.01) compared to the vehicle group. Other parameters remained stable.

Together, these data indicate that low-dose CBD co-therapy with CSA maintains a favourable safety profile in chronic colitis, while the olsalazine + CBD combination—despite mild elevations in some liver and kidney markers—corrects colitis-associated anaemia and thrombocytosis without overt haematological adverse effects.

### 2.10. Visual Summary of Results

**Figure 9:**
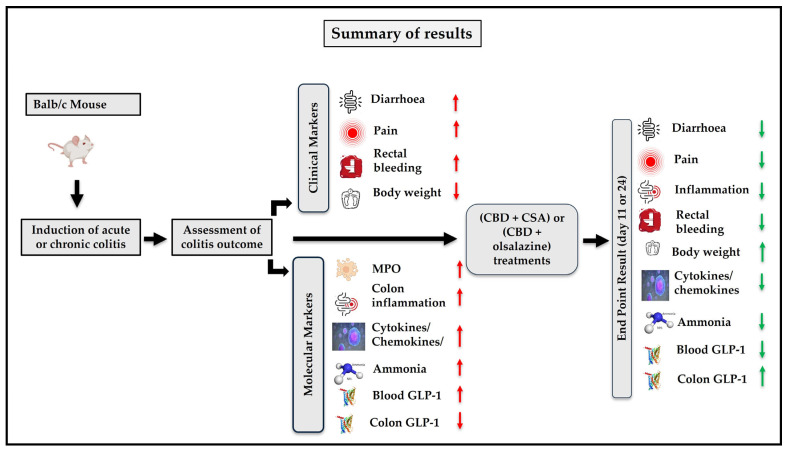
Schematic summary of the study design and outcomes showing the effects of combination treatments on clinical and molecular markers of colitis in Balb/c mice. Acute or chronic colitis was induced, followed by assessment of disease severity and response to treatment with either cannabidiol plus cyclosporine A (CBD + CSA) or cannabidiol plus olsalazine (CBD + olsalazine). Clinical markers (diarrhoea, pain, rectal bleeding, and body weight loss) and molecular markers (MPO activity, cytokines/chemokines, inflammation, ammonia, and GLP-1 levels) were evaluated. Red arrows (↑ or ↓) indicate dysregulation of markers in colitis, while green arrows represent correction or normalization following combination therapy.

Figure 9 provides a comprehensive visual summary of the experimental model, the key markers of colitis assessed, and the observed effects of the treatments on DAI scores, MPO activity, cytokine suppression, GLP-1 restoration, and safety-related endpoints.

## 3. Discussion

Current pharmacological treatments for inflammatory bowel disease often rely on high doses of anti-inflammatory or immunosuppressive agents, which, while effective, are frequently associated with significant limitations including increased risk of infections, systemic toxicity, loss of response, and difficulties in managing acute disease flares [28,44,45]. These adverse effects can compromise patient safety and limit long-term treatment adherence [46]. Cyclosporine A is primarily reserved for severe or steroid-refractory ulcerative colitis due to its potent immunosuppressive properties. However, its use is restricted by serious adverse effects, including nephrotoxicity, hypertension, and increased infection risk, making it unsuitable for long-term maintenance therapy [47,48]. Olsalazine and other 5-aminosalicylic acid (5-ASA) derivatives are generally better tolerated, yet high-dose regimens often provoke headaches, nausea, and rare hepatotoxicity, and many patients fail to achieve sustained remission on monotherapy [49,50]. Targeted therapies such as monoclonal antibodies and small molecules, while effective in managing moderate to severely active IBD, are expensive and not effective in all patients [51,52].

To address this gap, we hypothesized that low-dose combination therapies targeting complementary mechanisms could provide enhanced therapeutic outcomes while minimizing adverse effects. In this context, the combination of cannabidiol with conventional agents like cyclosporine A or olsalazine offers a promising alternative. CBD acts on multiple pharmacological targets, including CB1, CB2, GPR35, and peroxisome proliferator-activated receptors (PPARs), all of which are involved in inflammation and intestinal homeostasis, thereby exerting analgesic, anti-inflammatory, immunomodulatory, antipsychotic, and antimicrobial properties [18,19,53,54]. Notably, CBD has been shown to exert antimicrobial activity, particularly against gram-negative bacteria, through membrane disruption, as well as reduce biofilm formation and antibiotic resistance [55]. While CSA is associated with infection risk, and olsalazine requires high doses, the combination of sub-therapeutic doses of these agents with low-dose CBD would increase the effectiveness while also minimizing the chance of CSA-induced infection. Olsalazine also influences overlapping pathways with CBD, including NF-κB inhibition, oxidative stress modulation, and immune regulation [19,56,57]. Moreover, they may offer greater therapeutic flexibility for both maintenance and flare-phase treatment in IBD.

In this study, we comprehensively evaluated the therapeutic efficacy and safety of sub-therapeutic dose CBD in combination with cyclosporine A or olsalazine across both acute and chronic settings. Our findings consistently demonstrate that co-administration of sub-therapeutic doses of CBD enhances the effects of CSA or olsalazine in ameliorating clinical disease activity, preserving intestinal integrity, suppressing inflammatory mediators, and correcting metabolic and haematological disturbances. In the acute DSS model, CBD alone had a negligible impact on DAI or visceral pain, whereas CSA monotherapy reduced disease severity in a dose-dependent manner. Strikingly, the 10 mg/kg CBD + 5 mg/kg CSA combination significantly reduced DAI and grimace scores (Figure 1). A similar effect was observed with olsalazine: while monotherapies were not effective, the combination of 10 mg/kg CBD + 50 mg/kg olsalazine produced significant suppression of both DAI and visceral pain (Figure 1). Extending these combination regimens into a chronic colitis paradigm, both combinations maintained these benefits over repeated DSS cycles—sustaining low DAI scores, preventing weight loss, and reducing diarrhoea and faecal bleeding (Figure 2). The consistency between acute and chronic outcomes underscores the robustness of CBD’s potential to enhance the efficacy of CSA or olsalazine. These results suggest that CBD’s immunomodulatory and antioxidant properties synergize with CSA’s T-cell inhibition and olsalazine’s free-radical scavenging to amplify anti-inflammatory outcomes. These findings are in line with our previous findings wherein we reported enhanced effects of low-dose CBD (10 mg/kg) in combination with tetrahydrocannabinol (THC) or ZCZ011, a CB1 receptor allosteric modulator [43]. CBD on its own has also shown beneficial effects, but at a higher dose (60 mg/kg) [16].

The improvement in colon morphology and molecular markers readouts further confirmed clinical observations. CBD combinations fully prevented DSS-induced colon shortening and strongly suppressed MPO activity in colon tissue and spleen (Figure 3 and Figure 4). This suggests that CBD enhances mucosal protection and limits neutrophil infiltration to the colon and spleen. These findings are again in line with our previous findings, where CBD combination with THC and ZCZ011 restored colon length and MPO activity [43]. CBD alone also resulted in a similar response, but required higher doses [16,26]. CBD was also shown to directly reduce pain and neutrophil infiltration in an animal model of ocular pain and inflammation through modulation of the 5-hydroxytryptamine 1A (5-HT1A) receptor [58].

To further validate the efficacy of the combination treatments, we assessed the expression of key colonic inflammatory markers. In both acute and chronic colitis models, vehicle-treated mice showed a significant increase in pro-inflammatory cytokines, IL-6, IL-1β, MCP-1, and TNF-α, compared to healthy controls, which was markedly attenuated by the combination therapies (Figure 5 and Figure 6). Additionally, we investigated the expression of IL-17, a cytokine known for its strong pro-inflammatory properties and its pivotal role in the pathogenesis and persistence of IBD, in the chronic model [59]. Elevated IL-17 levels are a well-documented feature in individuals with active colitis [60]. Consistent with clinical observations, our chronic colitis model exhibited significantly increased IL-17 expression compared to healthy controls. Importantly, both combination treatments markedly reduced this elevation, underscoring their therapeutic potential in modulating key inflammatory pathways associated with chronic intestinal inflammation. While monotherapies like olsalazine often necessitate dose escalation to sustain therapeutic benefits—frequently accompanied by an increased risk of adverse effects [30,61]—our low-dose combination strategy achieved similar levels of mucosal healing and cytokine suppression to those observed with high-dose cyclosporine A, without the associated toxicity. Mechanistically, CBD likely enhances the efficacy of cyclosporine A and olsalazine through complementary modulation of overlapping anti-inflammatory pathways. By acting as a PPARγ agonist, CBD suppresses NF-κB activity and downstream cytokine production, mechanisms also engaged by 5-ASA compounds, thereby amplifying mucosal anti-inflammatory responses [62,63,64]. Concurrently, CBD’s activation of CB_2_ receptors on immune cells limits leukocyte infiltration and cytokine release, synergizing with CSA’s calcineurin/NFAT inhibition of T-cell activation [18,47,65,66]. CBD additionally inhibits NLRP3 inflammasome assembly and inducible nitric oxide synthase (iNOS) expression, reducing IL-1β and TNF-α levels [67,68], and promotes epithelial barrier integrity via PKA/AMPK signalling [69]. These convergent receptor-mediated actions allow for dose reductions of each drug while maintaining or improving therapeutic outcomes in colitis models. This broad control of both innate and adaptive inflammatory mediators aligns with the growing recognition that effective IBD management demands multi-targeted interventions rather than single-pathway blockade [22]. The current findings suggest that CBD’s immunomodulatory properties complement the established anti-inflammatory actions of CSA and olsalazine, converging on both innate and adaptive immune pathways to halt the cytokine cascade that drives mucosal injury.

Beyond classical colitis parameters, DSS exposure elicited pronounced metabolic disturbances, characterised by dysregulated GLP-1 signalling. This was further evidenced by hypoglycaemia, weight loss, and altered plasma and colonic GLP-1 concentrations (Figure 7 and Figure 8). GLP-1, an incretin hormone, despite its glucose regulatory and anti-inflammatory actions, is also implicated in several intestinal diseases, including IBD [41,70]. In acute colitis, we observed increased plasma and decreased colonic GLP-1 levels, consistent with our previous findings [16,43]. The loss of colonic L-cells with DSS likely explains reduced mucosal GLP-1, whereas systemic elevation may represent a compensatory mechanism to preserve gut integrity and limit inflammation [70]. Both combination therapies—cyclosporine A (CSA) + CBD and olsalazine + CBD—normalized blood glucose and mitigated weight loss. Notably, CSA + CBD selectively reduced plasma GLP-1 without altering colonic levels, while olsalazine + CBD robustly restored both plasma and colonic GLP-1, even exceeding healthy control values. In the chronic colitis model, olsalazine + CBD again improved glycaemic control and plasma GLP-1 but had no effect on colonic GLP-1; conversely, CSA + CBD increased colonic GLP-1 without impacting systemic levels or glucose. These differential patterns suggest that CSA + CBD may enhance local enteroendocrine function, whereas olsalazine + CBD exerts broader metabolic actions. Importantly, both regimens realigned gut hormonal signalling and improved overall metabolic homeostasis, highlighting a novel GLP-1–mediated axis for cannabinoid-based modulation in IBD. In addition to its role in glucose regulation, GLP-1 has been demonstrated to alleviate colitis in preclinical models [42,71].

A comprehensive safety evaluation (Table 1 and Table 2) demonstrated that the CSA + CBD combination was well tolerated, with no significant alterations observed in hepatic enzymes, renal function markers, or haematological parameters. Moreover, this regimen significantly ameliorated colitis-associated hyperammonemia—a systemic complication often linked to neurological disorders such as Alzheimer’s disease, depression, and anxiety, as well as hepatic dysfunction and gut microbiota dysbiosis, all of which represent recognized extraintestinal manifestations (EIMs) of inflammatory bowel disease (IBD) [22,72,73]. These findings suggest that, in addition to mitigating classical intestinal inflammation, the CSA + CBD combination may also confer therapeutic benefits for EIMs. In comparison, the olsalazine + CBD combination corrected anaemia and thrombocytosis but was associated with mild elevations in ALT, AST, and creatinine levels. Granulocyte count was slightly elevated in the vehicle group compared to the healthy control, although not statistically significant; this was significantly reduced by the olsalazine and CBD combination. Oral dosing shortly before euthanasia may explain the modest elevations in liver and kidney parameters observed with the cannabidiol–olsalazine combination, in contrast to the intraperitoneal (i.p.) CSA + CBD arm. In our prior work, i.p. CBD at 10 mg/kg did not adversely affect hepatic or renal function [16]. Although clinical olsalazine is generally well tolerated at doses up to 3 g/day, rare cases of interstitial nephritis and mild transaminase elevations have been reported with 5-aminosalicylates, suggesting the need for caution with higher doses [74,75]. It is therefore possible that a final oral dose on Day 24 induced transient hepatic or renal stress. To fully characterize safety, future studies should include alternative routes of administration, extended dosing regimens, comprehensive liver and kidney function panels, and histopathological evaluation of these organs. Nevertheless, the absence of overt haematological toxicity and the normalization of spleen and kidney weight indices in both treatment groups support the overall systemic safety of these therapeutic regimens.

While this study focused on female BALB/c mice, the therapeutic effects of CBD and its combinations have been documented in both sexes and across various mouse strains. For example, in male mice, CBD has been shown to ameliorate experimental colitis via immunomodulatory and antioxidant mechanisms [76]. Similarly, studies in male Swiss mice have demonstrated significant anti-inflammatory effects of CBD in models of intestinal and systemic inflammation [24]. Nonetheless, we acknowledge that sex and genetic background may influence pharmacodynamics and immune responses in the combination treatments. Future studies including both sexes, multiple strains, and alternative colitis models are warranted to fully explore the generalizability and translational relevance of the findings. Mechanistically, validation of CBD’s enhancement of cyclosporine A and olsalazine will require targeted interventions, such as receptor antagonists, RNA interference, or use of CB1-/CB2-, GPR35-, or PPAR-deficient models, to define the specific pathways involved. Although we demonstrated significant reductions in colonic cytokine levels, parallel analyses of circulating inflammatory markers would clarify systemic effects. Detailed characterization of immune cell subsets (e.g., Th1/Th17 cells and macrophages), alongside histopathological assessment of colonic crypt architecture, goblet cell preservation, and immune infiltration, will further elucidate the tissue-protective mechanisms. Future studies incorporating GLP-1 receptor (GLP-1R) antagonists or genetic knockout models will be critical to delineate the mechanistic contribution of GLP-1 signalling in mediating the observed therapeutic effects. These studies will be critical for translating our preclinical insights into safe, effective IBD therapies.

## 4. Materials and Methods

### 4.1. Animal Care and Use

All animal handling and experimental procedures adhered to the Australian Code for the Care and Use of Animals for Scientific Purposes and the New Zealand Guide for Animal Welfare in Research and Teaching. Ethical approval for the study was granted by the Curtin University Animal Research Ethics Committee (approval number: ARE2022-20). Female BALB/c mice aged 8–12 weeks (sourced from ARC and Ozgene, Perth, Australia) were used in this study following a one-week acclimatization period under specific pathogen-free (SPF) conditions. The environment was maintained with controlled temperature, humidity, and a 12-h light/dark cycle. Male mice were excluded from the study due to their propensity for cage-mate aggression, which could induce stress and confound experimental results. Mice were housed in groups of up to five per cage and provided with standard laboratory chow and water ad libitum.

### 4.2. Induction of Acute and Chronic Colitis

**Figure 10:**
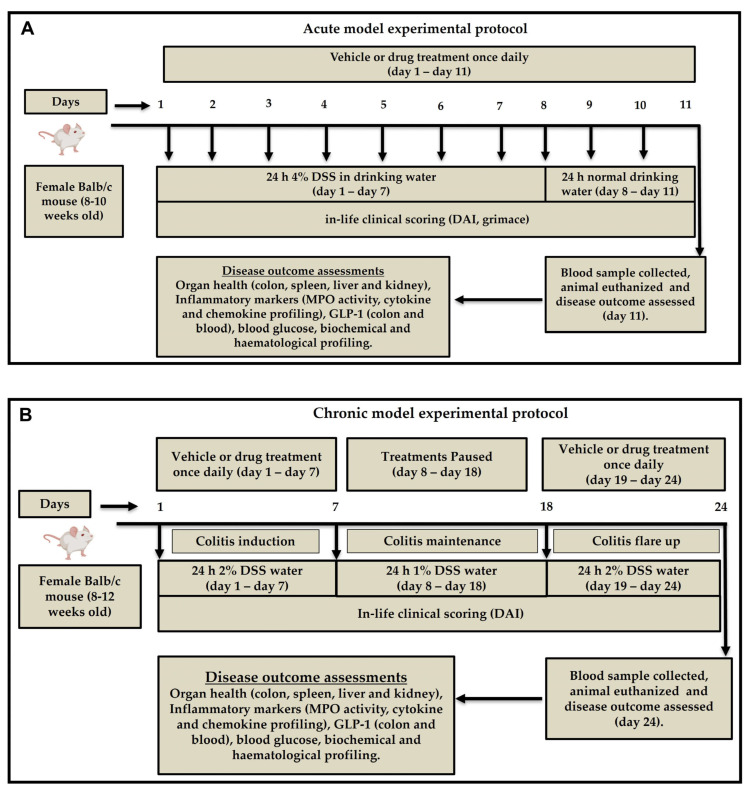
Experimental protocols for acute and chronic colitis models. (**A**) The acute model involved daily administration of vehicle or drug from days 1 to 11, with colitis induced by 4% dextran sulfate sodium (DSS) in drinking water from days 1 to 7, followed by 24 h of recovery. Disease activity was monitored via clinical scoring, and samples were collected on day 11. (**B**) The chronic model included three cycles of colitis induction using 2% DSS (flare) and 1% DSS (maintenance), with treatments administered on days 1–7 and 19–24. Clinical scoring was conducted throughout, and animals were euthanized on day 24 for outcome assessments. DAI—disease activity index; DSS—dextran sulfate sodium; GLP-1—glucagon-like peptide-1; MPO—myeloperoxidase.

Colitis-grade DSS (MW 36,000–50,000 Da, Lot No. S8634; MP Biomedicals, Santa Ana, CA, USA) was prepared at 4% (*w*/*v*) concentration in autoclaved drinking water. Acute and chronic colitis were induced as we previously reported [16]. Briefly, acute colitis was induced by administering 4% DSS solution to mice as the sole source of drinking water for seven consecutive days, followed by regular normal drinking water for three days. (Figure 10A) Chronic colitis was induced over 24 days. Mice were given 2% DSS for the first seven days, followed by 1% DSS for 10 days and 2% DSS for another seven days. Freshly prepared DSS solution is changed every three days for both acute and chronic models. Control mice in both models received normal drinking water throughout the experiment (Figure 10B).

### 4.3. Experimental Design and Pharmacological Treatments

Following 1 week of acclimatization at the research facility, mice cages were randomly assigned to various experimental groups, including healthy control, vehicle control, and drug treatment arms (*n* = 5–9). Group sizes were determined based on statistical power calculations and were consistent with those used in our previous study employing the same DSS-induced colitis model for exploration of other treatment strategies [16]. Briefly, a priori power analysis was conducted to justify the sample size of 5–9 animals per group. Effect sizes (Cohen’s *f*) for one-way ANOVA were calculated based on previously published data [43] for the Disease Activity Index (DAI) and cytokine measurements across three experimental groups. For DAI, group means, and standard deviations were 4.5 ± 0.53 (control), 0.125 ± 0.35 (treatment 1), and 0.375 ± 0.52 (treatment 2), resulting in an effect size of approximately 4.27. For cytokines, group means, and standard deviations were 6.05 ± 3.3 (control), 0.73 ± 0.19 (treatment 2), and 0.68 ± 0.21 (treatment 3), yielding an effect size of approximately 1.318. Based on these large effect sizes, the required sample size per group to achieve 80% power at a significance level (α) of 0.05 was estimated to be five animals. Therefore, the use of 5–9 animals per group in this study provides greater than 99% power to detect statistically significant differences, demonstrating that the chosen sample size is sufficiently powered for the analyses performed.

The pharmacological effects of cyclosporine A (CSA, Sandimmun^®^ 50 mg/mL; Novartis, Nuremberg, Germany), CBD (Commonwealth Extracts, Louisville, KY, USA), and olsalazine (Sigma-Aldrich, St. Louis, MO, USA) were evaluated in a DSS-induced colitis model, either as monotherapies or in combination. To minimize bias and ensure the integrity of the results, both treatment administration and data collection were conducted in a blinded manner. Mice were always treated in the same order, beginning with the vehicle control group. All compounds were formulated in a vehicle consisting of dimethyl sulfoxide (DMSO, 5%), Tween-80 (5%), and saline (90%) at a 1:1:18 ratio. Both DMSO and Tween-80 are widely used as vehicles in experimental research to improve the solubility of investigational agents. In our study, these vehicles were used at concentrations of 5%, which is generally considered safe on inflammatory responses in murine models as previously reported [77,78]. However, to minimise any potential confounding effects, we treated animals in the vehicle control group with an equal volume of the vehicle as the treatment arm.

In the acute colitis model, treatments were administered once daily from day 1 to day 11. In the chronic colitis model, treatments were given during 2% DSS exposure periods (days 1–7 and 18–24), with a drug-free recovery phase from day 8 to 17. All treatments were delivered at a volume of 10 mL/kg body weight. A sub-therapeutic dose of CBD at 10 mg/kg was selected based on previously published findings from our group, demonstrating limited efficacy when used alone [16]. The routes of administration were intentionally chosen according to the pharmacokinetic profiles and established mechanisms of action of each compound, as summarized in Table 3. Olsalazine is a prodrug designed to exert its therapeutic effects locally in the colon, where it is converted by colonic bacteria into its active form, 5-aminosalicylic acid (5-ASA). Oral administration is therefore essential for its efficacy. Intraperitoneal administration of olsalazine can bypass the gut lumen and prevent colonic activation of the drug. In contrast, cyclosporine A was administered i.p. to ensure consistent systemic bioavailability and avoid variability due to poor and unpredictable oral absorption, as well as significant first-pass metabolism, which are well-documented in rodent models [79,80]. CBD, used as an adjunct in both combinations, was delivered i.p. in the CSA arm and orally in the olsalazine arm to match the primary drug’s delivery route and optimize potential synergistic interactions. The treatment groups, routes of administration, number of animals, and observation periods for both acute and chronic models are summarized in Table 3. The most efficacious treatment identified in the acute model was subsequently evaluated in the chronic model.

### 4.4. Assessment of the DAI Score

As previously reported by our group [16], the DAI score was calculated by summing three primary clinical parameters: body weight loss, diarrhoea/stool consistency, and the presence of occult blood or rectal bleeding (Table 4). The parameters of DAI, including body weight loss, diarrhoea, and faecal blood/rectal bleeding, were assessed daily (9 AM–11 AM) post-DSS administration to monitor colitis progression and treatment effects throughout the study. Data collection was performed using a standardized paper-based scoring sheet designed specifically for this study, ensuring consistent and accurate recording of individual animal scores at each time point. Faecal blood or rectal bleeding was assessed by visual inspection of stool and the perianal area during daily monitoring. These assessment methods were validated by the veterinarian at the animal research facility to ensure accuracy and reliability.

### 4.5. Pain Behaviours

Small laboratory animals often exhibit pain through facial expressions, making it challenging to standardize observations of pain behaviours. To address this, the Grimace Scoring Method (GSM), a reliable and non-invasive approach, was employed in this study to evaluate pain behaviours [81]. Mice were observed daily for five specific facial features associated with discomfort: orbital tightening, nose bulge, whisker retraction, cheek bulge, and changes in ear position. In addition, alterations in body posture and movement were monitored as general indicators of pain or distress. Grimace scoring for visceral pain was conducted concurrently with DAI assessments, following established protocols (Table 5). All observations and score calculations were performed by trained personnel blinded to treatment groups. The following standardized criteria were applied to assess pain-related behaviours in the mice.

### 4.6. Random Blood Glucose

The random glucose level (mmol/L) was measured in tail-pricked blood using a handheld blood glucometer (model) on day 11 or day 24 across all experimental groups. This measurement was conducted to assess whether DSS-induced colitis and the treatments had any effect on random blood glucose levels.

### 4.7. Euthanasia and Tissue Collection

On day 11 (acute model) or day 24 (chronic model), the endpoint of the experiments, mice were anesthetized with 2.5–3% isoflurane in the presence of oxygen (L/min), and blood was collected via cardiac puncture. After blood collection, the mice were euthanized by cervical dislocation. Colon, spleen, liver, and kidney tissues were then harvested, cleaned with phosphate-buffered saline (PBS), and assessed for various markers of inflammation.

### 4.8. Colon Length

Colon length measurement is a critical macroscopic parameter for evaluating the severity of colonic inflammation and damage in a DSS-induced colitis mouse model, as colitis in both humans and animals is associated with a significant reduction in colon length. To assess the potential therapeutic effects of treatments, the colon was excised from the cecum to the rectum post-euthanasia, placed on a clean surface, and measured from the cecal tip to the rectum using a ruler under consistent conditions, with images captured for documentation. Following measurement, the colon was washed thoroughly with 1% cold PBS, divided longitudinally into two halves, and processed for further analysis: one half was snap-frozen in liquid nitrogen and stored at −80 °C for molecular studies, while the other was fixed in formalin for histological evaluation.

### 4.9. Organ-to-Body Weight Percentage

Colitis is often accompanied by extraintestinal manifestations in addition to colon inflammation. To evaluate the potential impact of colitis on other organs, vital organs including the liver, spleen, and kidneys were carefully excised, gently dried with paper towels, and weighed. The relative weight of each organ was calculated as a percentage of the animal’s body weight and subsequently compared and analysed across experimental groups.

### 4.10. Myeloperoxidase (MPO) Activity

MPO, an enzyme primarily found in neutrophilic granulocytes, plays a critical role in the immune response and serves as a marker of acute inflammation. Its activity reflects the presence and activation of neutrophils, which are key players in inflammation. Measuring MPO levels in colitis provides valuable insights into the severity of inflammation and the efficacy of anti-inflammatory treatments.

To assess MPO activity in colon and spleen tissues, a standardized protocol, as we previously reported, was followed. Tissue samples were collected, dried, weighed, and homogenized in a potassium phosphate buffer containing HTAB. After centrifugation, the supernatant was mixed with a buffer containing O-dianisidine hydrochloride and hydrogen peroxide, and the reaction kinetics were measured spectrophotometrically at 460 nm. MPO activity was quantified as a percentage relative to the healthy control group (set at 100%), with results for treatment groups expressed as a percentage change compared to the vehicle-treated group.

### 4.11. Colon Cytokine and Chemokine Quantification

Pro-inflammatory cytokines, including TNFα, interleukin-1 beta (IL-1β), interleukin-6 (IL-6), interleukin-12 (IL-12), interferon-gamma (IFN-γ), chemokine ligand 2 (CCL2), and anti-inflammatory cytokine interleukin-10 (IL-10), were quantified in colon tissues using a multiplex assay kit (MCYTOMAG-70K, Merck Millipore, Darmstadt, Germany). The assay was performed according to the manufacturer’s instructions. Briefly, frozen colon tissues stored at −80 °C were homogenized in radioimmunoprecipitation assay (RIPA) buffer (Sigma-Aldrich, St. Louis, MO, USA), sonicated, and centrifuged at maximum speed for 15 min. The supernatant was collected, and protein concentration was measured using a Micro bicinchoninic acid (BCA) assay (Thermo Fisher Scientific, Waltham, MA, USA). Cytokine and chemokine concentrations were quantified using a MagPix Luminex 100 reader (Luminex Corporation, Austin, TX, USA), and results were expressed in picograms per milligram (pg/mg) of protein.

### 4.12. GLP-1 Measurement

Plasma and colon GLP-1 levels were measured using a commercially available ELISA kit (EMD Millipore, Darmstadt, Germany). The assay was performed according to the manufacturer’s instructions, and readings were obtained using a multimode 96-well plate reader. Results were expressed as picomolar (pmol) for plasma samples and pmol/µg of protein for colon lysates.

### 4.13. Haematology and Biochemistry

Fresh whole blood in heparin-coated tubes was analysed for haematological parameters using an automated blood analyser (BC-2800, Mindray, Shenzhen, China). Biochemical markers (ALT, AST, BUN, albumin, globulin, creatinine, ammonia, and total protein) were measured in plasma using an automated biochemistry analyser (Element DC, Heska, Loveland, CO, USA).

### 4.14. Statistical Analysis

Data are presented as mean ± standard error of the mean (SEM). For the acute colitis study, statistical comparisons among all treatment groups were performed using one-way ANOVA followed by Tukey’s multiple comparisons test. In the chronic colitis study, one-way ANOVA followed by Dunnett’s multiple comparisons test was used to compare each treatment group directly with the vehicle control group. For daily measurements, including DAI, grimace score, and body weight, a two-way ANOVA with Dunnett’s multiple comparisons test was applied to assess treatment effects over time in both the acute and chronic models, as previously reported in similar experimental settings [43,82]. Statistical significance is represented as follows: a hash symbol (#) indicates a significant difference between healthy controls and the vehicle-treated colitis group, while an asterisk (*) denotes a significant difference between drug-treated groups and the vehicle-treated colitis group. A *p*-value of less than 0.05 (*p* < 0.05) was considered statistically significant. All analyses were conducted using GraphPad Prism (version 8; GraphPad Software, San Diego, CA, USA).

## 5. Conclusions

Collectively, our data provide a strong preclinical rationale for leveraging low-dose CBD to enhance the efficacy of existing IBD therapies. The reproducible synergistic effects observed in acute and chronic colitis models—spanning clinical, morphological, molecular, metabolic, and safety domains—underscore CBD’s potential as a safer adjunct agent. CBD co-therapy with CSA or olsalazine offers a multifaceted approach to IBD treatment—achieving superior disease suppression, preserving intestinal and systemic homeostasis, and maintaining an acceptable safety profile. This strategy holds promise for improving patient outcomes while potentially reducing the doses and side effects of conventional IBD drugs. Clinical trials will be essential to confirm safety and efficacy in human IBD patients.

## Figures and Tables

**Table 1:** Effect of treatments on blood biochemistry and relative organ weight in DSS-induced chronic colitis.

Biochemical Parameters and Relative Organ-Body Weight	Healthy Control	Vehicle	10 mg/kg CBD + 5 mg/kg CSA	10 mg/kg CBD + 50 mg/kg Olsalazine
ALT	31.3 ± 1.6	36.4 ± 3.6	41.6 ± 2.9	63.9 ± 9.6 **
AST	75.0 ± 7.1	66.3 ± 5.7	55.0 ± 6.9	120.1 ± 17 **
BUN	6.2 ± 0.2	6.1± 0.3	5.2± 0.4	5.9 ± 0.2
Creatinine	18.5 ± 0.3	22.9 ± 1.3 ^#^	20.0 ± 0.7	28.7 ± 2.1 **
TP	44.8 ± 0.9	41.0 ± 0.9	38.0 ± 1.9	43.4 ± 0.8
Albumin	22.5 ± 0.5	19.9 ± 0.4 ^##^	19.3 ± 0.7	21.5 ± 0.5
Globulin	22.3 ± 0.7	21.1 ± 1.0	18.8 ± 1.5	22.0 ± 0.9
Albumin/Globulin	1.025 ± 0.031	0.963 ± 0.063	1.075 ± 0.1	1.0 ± 0.1
Ammonia	40.8 ± 1.9	76.5 ± 6.9 ^####^	50.6 ± 5.6 **	43.9 ± 4.0 ***
Relative liver-body weight (%)	4.1 ± 0.05	4.7 ± 0.1 ^###^	4.5 ± 0.05	4.1 ± 0.1 ***
Relative kidney-body weight (%)	1.001 ± 0.011	1.064 ± 0.02 ^#^	1.052 ± 0.02	1.001 ± 0.01 *

Data are presented as mean ± SEM; statistically significant differences were calculated using one-way ANOVA followed by Dunnet’s multiple comparisons test and are indicated by ^#^ *p* < 0.05, ^##^ *p* < 0.01, ^###^ *p* < 0.001, ^####^ *p* < 0.0001 compared to healthy control; and * *p* < 0.05, ** *p* < 0.01, *** *p* < 0.001 compared to vehicle.

**Table 2:** Effect of treatments on blood haematology in DSS-induced chronic colitis.

Haematological Parameters	Healthy Control	Vehicle	10 mg/kg CBD + 5 mg/kg CSA	10 mg/kg CBD + 50 mg/kg Olsalazine
WBC (10^9^/L)	6.30 ± 0.91	6.71 ± 0.99	4.79 ± 0.62	4.89 ± 0.56
Lymphocytes (10^9^/L)	3.10 ± 1.13	2.32 ± 0.94	2.34 ± 1.03	3.48 ± 0.58
Monocytes (10^9^/L)	0.23 ± 0.08	0.32 ± 0.14	0.19 ± 0.05	0.24 ± 0.04
Granulocytes (10^9^/L)	3.00 ± 0.76	4.06 ± 0.76	2.28 ± 0.54	1.21 ± 0.19 **
RBC (10^12^/L)	9.28 ± 0.11	8.77 ± 0.11 ^#^	8.83 ± 0.15	9.44 ± 0.10 ***
Haemoglobin (g/L)	135.88 ± 1.87	128.10 ± 2.02 ^#^	131.00 ± 2.80	136.75 ± 1.92 *
Haematocrit (%)	42.39 ± 0.54	40.00 ± 0.64 ^#^	38.89 ± 0.63	42.39 ± 0.66 *
MCV (fL)	45.66 ± 0.20	45.60 ± 0.35	44.13 ± 0.07	44.78 ± 0.23
MCH (pg)	14.58 ± 0.08	14.60 ± 0.10	14.79 ± 0.09	14.42 ± 0.08
MCHC (g/L)	320.25 ± 2.52	320.00 ± 1.69	336.63 ± 2.04	321.88 ± 1.17
RDW (%)	11.66 ± 0.14	12.47 ± 0.24	12.28 ± 0.15	12.02 ± 0.11
Platelets (10^9^/L)	1006.71 ± 56.06	974.0 ± 35.51	876.00 ± 49.56	1221.1 ± 72.94 **
MPV (fL)	4.88 ± 0.04	4.89 ± 0.06	4.71 ± 0.07	4.76 ± 0.17
PDW	16.30 ± 0.05	16.22 ± 0.05	16.00 ± 0.04	16.26 ± 0.20
Procalcitonin (%)	0.50 ± 0.03	0.50 ± 0.02	0.40 ± 0.02	0.45 ± 0.05

Data are presented as mean ± SEM; statistically significant differences were calculated using one-way ANOVA followed by Dunnett’s multiple comparisons test and are indicated by ^#^ *p* < 0.05 compared to healthy control; and * *p* < 0.05, ** *p* < 0.01, *** *p* < 0.001 compared to vehicle.

**Table 3:** Summary of treatment groups, route of administration, animal numbers, and observation periods in acute and chronic colitis models.

Acute Model (11 Days)				Chronic Model (24 Days)	
CSA cohort (i.p. route of administration)		Olsalazine cohort (oral route of administration)		Treatment groups (oral route of administration)	Number of animals
Treatment groups	Number of animals	Treatment groups	Number of animals		
Healthy control	8	Healthy control	8	Healthy control	8
Vehicle	8	Vehicle	8	Vehicle	9
2.5 mg/kg cyclosporine	5	10 mg/kg CBD	8	10 mg/kg CBD + 5 mg/kg cyclosporine	8
5 mg/kg cyclosporine	5	50 mg/kg Olsalazine	5	10 mg/kg CBD + 50 mg/kg olsalazine	8
10 mg/kg cyclosporine	5	10 mg/kg CBD + 50 mg/kg olsalazine	8		
10 mg/kg CBD	5				
2.5 mg/kg cyclosporine + 10 mg/kg CBD	8				
5 mg/kg cyclosporine + 10 mg/kg CBD	8				

**Table 4:** Assessment of DAI score.

Parameter	Score 0	Score 1	Score 2	Score 3
Body weight loss	<5%	5–10%	11–15%	16–20%
Diarrhoea/stool consistency	Normal	Mild-soft, but still formed	Very soft/Sticky	Loose/Diarrhoea
Faecal blood/rectal bleeding	Normal colour stool	Brown colour stool	Darker reddish colour stool	Perianal blood/rectal bleeding

**Table 5:** Assessment of grimace score.

Pain Features/Scores	0	1	2
Orbital tightening	Not present	Closing of the eyelid, narrowing of the orbital area	Complete closure of the eye with tightened orbital
Nose bulge	Not present	Slight bulging on the bridge of the nose	Completely bulged nose
Cheek bulge	Not present	Slight bulging of the cheek	Completely bulged
Ear position	Normal position	Ears moving towards the back	Folded ear forming a pointed shape
Whisker change	Normal whisker position	Whiskers pulled back/front	Clumping of whiskers
Movement/gait	Normal activity	Moves slowly	Moves only when provoked
Body position/hunching	Normal position	Slight tuck to the abdomen	Fully hunched

The grimace score is calculated as the sum of all 7 pain behaviours.

## Data Availability

The original contributions presented in this study are included within the article. Additional data will be made available upon request, and any further inquiries should be directed to the corresponding authors.

## Abbreviations

The following abbreviations are used in this manuscript:

**Abbreviation**	**Full Form**
5-ASA	5-Aminosalicylic Acid
5-HT1A	5-Hydroxytryptamine 1A receptor
ALT	Alanine Aminotransferase
AMPK	AMP-Activated Protein Kinase
AST	Aspartate Aminotransferase
BCA	Bicinchoninic Acid
BUN	Blood Urea Nitrogen
CB1	Cannabinoid Receptor Type 1
CB2	Cannabinoid Receptor Type 2
CBD	Cannabidiol
CCL2	Chemokine (C-C motif) Ligand 2
CD	Crohn’s Disease
CSA	Cyclosporine A
DAI	Disease Activity Index
DMSO	Dimethyl Sulfoxide
DSS	Dextran Sulphate Sodium
ECS	Endocannabinoid System
EIMs	Extraintestinal Manifestations
GLP-1	Glucagon-Like Peptide-1
GPR35	G Protein-Coupled Receptor 35
GSM	Grimace Scoring Method
IBD	Inflammatory Bowel Disease
IFN-γ	Interferon-gamma
IL-10	Interleukin-10
IL-12	Interleukin-12
IL-17	Interleukin-17
IL-1β	Interleukin-1 beta
IL-6	Interleukin-6
MAPK	Mitogen-Activated Protein Kinase
MCH	Mean Corpuscular Haemoglobin
MCHC	Mean Corpuscular Haemoglobin Concentration
MCP-1	Monocyte Chemoattractant Protein-1
MCV	Mean Corpuscular Volume
MPO	Myeloperoxidase
MPV	Mean Platelet Volume
NF-κB	Nuclear Factor kappa-Light-Chain-Enhancer of Activated B cells
NFAT	Nuclear Factor of Activated T-Cells
NLRP3	NOD-, LRR-, and Pyrin Domain-Containing Protein 3 (inflammasome)
PDW	Platelet Distribution Width
PKA	Protein Kinase A
PPAR	Peroxisome Proliferator-Activated Receptor
RBC	Red Blood Cell
RDW	Red Cell Distribution Width
RIPA	Radioimmunoprecipitation Assay
SPF	Specific Pathogen-Free
T-Cell	T Lymphocyte
THC	Tetrahydrocannabinol
TNF-α	Tumour Necrosis Factor-alpha
TP	Total Protein
UC	Ulcerative Colitis
WBC	White Blood Cell
eCBome	Endocannabinoidome
i.p.	Intraperitoneal
iNOS	Inducible Nitric Oxide Synthase
